# Scale-Up Production of Type O and A Foot-and-Mouth Disease Bivalent Vaccine and Its Protective Efficacy in Pigs

**DOI:** 10.3390/vaccines9060586

**Published:** 2021-06-02

**Authors:** Sang-Hyun Park, Seo-Yong Lee, Jae-Seok Kim, Ah-Young Kim, Sun-Young Park, Ji-Hye Lee, Mijung Lee, Hyejin Kim, Sim-In Lee, Na-Young Kang, Jung-Won Park, Su-Mi Kim, Jong-Hyeon Park, Young-Joon Ko

**Affiliations:** 1 Animal and Plant Quarantine Agency, 177 Hyeoksin-8-ro, Gimcheon-si 39660, Gyeongsangbuk-do, Korea; shpark0205@korea.kr (S.-H.P.); silveryfox2@naver.com (J.-S.K.); mochsha@korea.kr (A.-Y.K.); sun3730@korea.kr (S.-Y.P.); hyejin86@korea.kr (H.K.); lunark2@korea.kr (S.-I.L.); shdud9111@naver.com (N.-Y.K.); parkjw6254@korea.kr (J.-W.P.); beliefsk@korea.kr (S.-M.K.); parkjhvet@korea.kr (J.-H.P.); 2 Research Unit, FVC Vaccine Company, #521, 5, Hyeoksin 8-ro, Gimcheon-si 39660, Gyeongsangbuk-do, Korea; sylee@gcfvc.com (S.-Y.L.); jhlee@gcfvc.com (J.-H.L.); mjlee@gcfvc.com (M.L.)

**Keywords:** FMD, foot-and-mouth disease, scale-up, bivalent vaccine, protective efficacy

## Abstract

South Korea has experienced FMD outbreaks almost every year since 2014. Therefore, a novel local vaccine that can cover various topotypes of viruses is required. Two virus strains, O/Boeun/SKR/2017 and A/Yeoncheon/SKR/2017, were cultured up to the pilot scale based on the optimized conditions set up on the flask scale. FMDV particles (146S) of 2 µg/mL or more were obtained from the virus culture supernatant using a 100 L bioreactor. The viruses were fully inactivated using binary ethylenimine within 16 h through two inactivation cycles and mixed with an adjuvant into a bivalent vaccine (types O and A) consisting of 15 µg viruses per strain. The experimental bivalent vaccine showed a broad spectrum of high neutralizing antibody titers against heterologous viruses, including type O Cathay strain and type A Asia topotypes, except for GVII. The 50% protective dose was determined as 12.5 for O/Boeun/SKR/2017 and 15.6 for A/Yeoncheon/SKR/2017. Collectively, we expect that the bivalent vaccine could protect against FMDV types O and A circulating in South Korea and neighboring countries. To our knowledge, this is the first report demonstrating that the vaccine strains could be successfully scaled-up to a 100 L bioreactor, with the determination of its protective efficacy in pigs.

## 1. Introduction

Foot-and-mouth disease (FMD) is a highly contagious disease that affects cloven-hoof animals, including pigs, cattle, goats, and sheep, causing severe economic damage to the livestock industry [1]. The causative agent, FMD virus (FMDV), belongs to the *Picornaviridae* family. There are seven serotypes of FMDV (O, A, Asia 1, C, SAT-1, SAT-2, and SAT-3), and the rapid mutation rate results in numerous variants in the same serotype with no protective cross-reactivity [2].

The serotype O FMDV consists of 10 topotypes. Of these, three distinct topotypes are recognized in Southeast Asia (SEA): O/SEA, O/Middle East-South Asia (ME-SA), and O/Cathay [3]. The SEA topotype (O/SEA/Mya-98) strain spread to South Korea and Japan during 2010–2011. In 1999–2000, the O/ME-SA/PanAsia lineage was introduced into SEA, causing extensive outbreaks in Japan, South Korea, China, and Russia [4]. The O/ME-SA/PanAsia lineage is divided into PanAsia and PanAsia-2, with the latter being reported only in SEA in 2003–2009 [4]. More recently, the ME-SA/Ind-2001 originating from India spread to North Africa, the Middle East, SEA, and the Far East between 2013 and 2017 [4]. The O/Cathay, characterized by a deletion within its 3A region, was identified in 2001 [5]. This topotype is currently endemic in SEA; thus, it has the potential to be introduced into South Korea.

FMD serotype A viruses consist of three major topotypes: Euro-SA, Asia, and Africa. All serotype A viruses found in SEA belong to the Asian topotype. The Asia topotype comprises the A15, A22, Iran-05, Thai-87, SEA-97, and G-VII lineages [6].

Although all FMD-susceptible livestock were vaccinated in South Korea, the FMD outbreak has continued almost every year since 2014. There were two outbreaks due to serotype O SEA topotypes of FMDV in 2014–2015 and one in 2016. Although no further SEA topotype of FMD has been reported in Korea since then, new outbreaks of the ME-SA/Ind-2001 lineage virus emerged in 2017 and 2019. Additionally, new topotypes of FMDV serotype O, such as Cathay, have been reported in other countries [7]. There were two outbreaks of serotype A in South Korea in 2010 and 2017. The causative serotype A viruses belong to A/ASIA/SEA-97.

In this regard, it is necessary to develop a new bivalent vaccine with a broad spectrum of protection against many topotypes of FMDV for serotypes O and A. Thus, this study aimed to produce vaccine antigens of serotype O and A up to pilot scale (100 L), and to determine the protective efficacy in pigs before local vaccine production on an industrial scale.

## 2. Materials and Methods

### 2.1. Cells

The cell line “KCTC 12945BP” that was developed from the original adherent cell line, BHK-21 [C-13] (ATCC, Manassas, VA, USA), by the Animal and Plant Quarantine Agency (APQA) and the Korea Research Institute of Bioscience & Biotechnology for use in suspension culture with serum-free media, was adapted for growth in Cellvento^TM^ BHK-200 cell culture medium (Merck, Darmstadt, Germany) by incubation at 110 rpm in a shaking incubator at 37 °C with 5% CO_2_. Cell numbers and viability were analyzed via the trypan blue exclusion method using an automated cell counter (Vi-Cell XR; Beckman Coulter Inc., Brea, CA, USA). A 70% volume of the total cells with 3 × 10^5^ cells/mL was grown for 3.5 days up to approximately 3 × 10^6^ cells/mL, and 30% volume of fresh Cellvento media was added without removing the spent media, followed by inoculation of FMDV into a flask or bioreactor. 

### 2.2. Viruses

O/Boeun/SKR/2017 (GenBank accession No. MG983730.1) and A/Yeoncheon/SKR/2017 (GenBank accession No. KY766148.1) were isolated by APQA during FMD outbreaks in Korea and adapted to BHK-21 suspension cells [8]. The other viruses used for the virus neutralization test were O/Jincheon/SKR/2014 (GenBank accession No. 162590.1), O/Gimje/SKR/2016 (GenBank accession No. KY086465.1), O/Anseong/SKR/2015 (GenBank accession No. KU991734.1), Taiwan 97 (GenBank accession No. KJ831708.1), O/VIT/2013 (GenBank accession No. KY492067.1), A/Pocheon/SKR/2010 (GenBank accession No. KC588943.1), A/Gimpo/SKR/2018 (GenBank accession No. MK463492.1), A22 Iraq/24/64 (GenBank accession No. AY593764.1), and A/Nepal/12/2017. O Taiwan 97 was obtained from the Pirbright Institute (OIE/FAO reference laboratory for FMD, Woking, UK). A22 Iraq/24/64, a recombinant virus, was prepared as described by Lee et al. [9]. O/Jincheon/SKR/2014, O/Gimje/SKR/2016, O/Anseong/SKR/2019, A/Pocheon/SKR/2010, and A/Gimpo/SKR/2018 were isolated by APQA and O/VIT/2013 was isolated by APQA using field samples supplied by the National Center for Veterinary Diagnosis in Vietnam. The phylogenetic tree was constructed based on the VP1 sequence. The genetic relationship was assessed through the neighbor-joining method using MEGA X software with 1000 replicates of bootstrap analysis.

### 2.3. Determination of Optimal Conditions to Propagate FMDV on a Flask Scale

When the number of BHK-21 suspension cells reached approximately 3 × 10^6^ cells/mL after 3.5 days, the FMDV O/Boeun/SKR/2017 or FMDV A/Yeoncheon/SKR/2017 was inoculated onto the cells at a multiplicity of infection (MOI) of 0.0005, 0.001, 0.005, 0.01, and 0.05 in a shaking incubator at 37 °C with 5% CO_2_. Viruses were harvested at 8, 12, 16, 20, and 24 h post-infection (hpi), and clarified by centrifugation at 3000× *g* for 20 min at 4 °C to remove cell debris. The virus titer and 146S antigen were measured as described in Section 2.5 and Section 2.7, respectively.

### 2.4. Production of FMD Vaccine Antigens on a Pilot Scale 

After Cellvento BHK-200 cell culture media were prepared in a mixer (Sartorius, Goettingen, Germany), 70 L of media was transferred to a 100 L bioreactor (Sartorius) with an initial cell density of 3 × 10^5^ cells/mL. The bioreactor was equipped with probes to measure and control the temperature and dissolved oxygen at 37 °C and 50% air saturation. The pH was controlled in the range of 7.2–7.4 by the addition of CO_2_ and 1 M NaOH solution. The agitation rate was maintained at 100 rpm. Samples were taken every 24 h to evaluate the cell density and viability. After the cell density reached approximately 3 × 10^6^ cells/mL, 30 L of the Cellvento BHK-200 cell culture medium was added to the bioreactor. The FMDV O/Boeun/SKR/2017 or A/Yeoncheon/SKR/2017 was also added together with the fresh media in the bioreactor at 0.001 and 0.0005 MOI, respectively. The virus culture supernatants were subsequently harvested at 16 and 12 hpi, respectively. The virus culture supernatants were transferred to a 250 L inactivation tank (BT Resources, Gyeonggi, Korea) and inactivated with 3 mM binary ethyleneimine (BEI, Sigma-Aldrich, St. Louis, MO, USA) through a consecutive inactivation process for 24 h at 26 °C using two individual tanks. Samples were taken every hour from the inactivation tank for 6 h to measure virus inactivation kinetics for each strain of the virus. The extrapolation of the individual graph was drawn as a linear line to analyze the FMDV inactivation kinetics. 

The inactivated viruses were treated with 50% Polyethylene Glycol (PEG) 6000 (Sigma-Aldrich) into a final 7.5% PEG for 16 h and harvested through a continuous centrifuge (Tomoe, Tokyo, Japan) at 16,400 rpm and a flow rate of 42 L/h. The pellet was resuspended in 50 mM Tris containing 300 mM KCl (pH 7.6) and stored at −70 °C until use. 

### 2.5. Virus Titration

Virus titers were determined with the adherent BHK-21 cells via an endpoint titration using the Spearman-Kärber calculation and presented as the tissue culture infectious dose affecting 50% of the cultures (TCID_50_) per mL [10,11].

### 2.6. Virus Neutralization Test

The virus neutralization (VN) test was performed using the methods described in the OIE terrestrial manual [12]. The sera were inactivated at 56 °C for 30 min before testing. Fifty microliters of two-fold serially diluted sera starting from 1/4 dilution were mixed with 50 μL of each virus containing 100 TCID_50_. After incubation at 37°C for 1 h, 50 μL of fetal porcine kidney (LFBK, supplied by Plum Island Animal Disease Center (Orient, NY, USA)) cells (0.5 × 10^6^ cells/mL) were added to each well. Plates were sealed and incubated in an incubator at 37 °C with 5% CO_2_ for 2–3 days. The VN titer was determined according to the Spearman–Kärber method and expressed as a log_10_ value.

### 2.7. Quantification of Vaccine Antigen (146S) 

The harvested virus culture medium was treated using chloroform (Merck KGaA, Darmstadt, Germany) 1:1 (*v/v*) and mixed by vigorous inversion for 5 min. The mixture was centrifuged at 3000× *g* for 15 min at 4 °C, and the aqueous layer on top of the organic solvent was harvested. Thereafter, these samples were treated with benzonase (Merck KGaA) at a concentration of 25 units/mL and incubated at 37 °C for 1 h. After centrifugation at 10,000× *g* for 30 min, the supernatant was harvested. The pretreated samples were layered onto 15–45% sucrose density gradients and ultracentrifuged at 100,000× *g* for 4 h at 4 °C using an SW41Ti rotor. The ultracentrifuged gradient tube was fractionated using a continuous density gradient fractionator (Teledyne ISCO, Lincoln, NE, USA), and the absorbance of each fraction at 254 nm was recorded using the spectrophotometer component of the instrument. The area under the peak for specific fractions was measured to calculate the quantity of 146S antigens (µg/mL) according to the formula presented in a previous study [13].

### 2.8. Transmission Electron Microscopy

The viral suspension that was concentrated using Polyethylene Glycol (PEG) was layered on top of sucrose gradients and ultracentrifuged at 100,000× *g* for 4 h. The band between the 30–35% sucrose layers was collected and ultracentrifuged at 100,000× *g* for 4 h. The resulting pellet was resuspended and dialyzed using 50 mM Tris containing 300 mM KCl (pH 7.6) to eliminate residual sucrose at 4 °C. One drop of the purified FMDV suspension was placed on Formvar-coated grids and negatively stained with 1% uranyl acetate. The FMDV particles were examined using transmission electron microscopy (H-7100FA; Hitachi, Tokyo, Japan). 

### 2.9. Immune Responses in Guinea Pigs Vaccinated with the Bivalent Vaccine

The antigens for type O and A, as described in Section 2.7, were mixed with 1% saponin (Sigma-Aldrich) and 10% aluminum hydroxide gel (General Chemical, NJ, USA) to prepare a bivalent vaccine. The ISA 206 VG adjuvant (Seppic, Paris, France) pre-warmed at 30 °C was then added at a ratio of 1:1, resulting in 1 mL/dose or 2 mL/dose including 15 µg of the types O and A. After the mixtures were incubated at 20 °C for 1 h in a water bath without light exposure, they were stored at 4 °C until use. A total of 10 guinea pigs weighing 230–250 g was divided into two groups. Animals in the first group (*n* = 5) were inoculated intramuscularly at a volume of 1/10 of 1 mL/dose, and those in the other group (*n* =5) were inoculated intramuscularly at a volume of 1/10 of 2 mL/dose. Blood samples of each guinea pig were collected at 0, 7, 14, 21, 28, 56, and 84 days post-vaccination (dpv). VN titers with sera at 28 dpv were investigated against homologous and heterologous viruses using several topotypes of viruses. For serotype O viruses, O/Gimje/SKR/2016 and O/Jincheon/SKR/2014 belong to the SEA topotypes. The two ME-SA topotypes of the viruses were O/Anseong/SKR/2019 and O/VIT/2013. Cathay’s topotypes of the viruses were Taiwan 97. For serotype A viruses, the A/ Pocheon/SKR/2010, A/Gimpo/SKR/2018 (SEA-97), A22 Iraq (A22), and A/Nepal/12/2017 (G-VII) belong to Asia topotypes (Figure S1). 

### 2.10. Vaccine Potency Test in Pigs

Two-month-old growing pigs (*n* = 18) who were not previously vaccinated with FMD, were divided into four groups. Pigs in the first group (five heads per group) were immunized with a full dose of bivalent vaccine containing 15 µg/mL of 146S for each strain (1 mL). The second and third groups were immunized with the same vaccine at 1/3 dose (0.33 mL) and 1/9 dose (0.11 mL), respectively. The control group consisted of three pigs without vaccination. Blood samples were collected at 0, 7, 14, 21, and 28 dpv. Pigs in all groups were challenged with O/Boeun/SKR/2017 or A/Yeoncheon/SKR/2017 at 28 dpv at 1 × 10^5^ TCID_50_/0.1 mL in the bulb of the heel. When clinical signs of FMD were observed, they were immediately isolated to prevent a second viral infection. The clinical score was calculated using the following criteria: reduced appetite (1 point) or no food intake and food leftover (2 points); lameness (1 point) or reluctance to stand (2 points); the presence of heat and pain after palpation of the coronary band (1 point) or not standing on the affected foot (2 points); vesicles on the foot, dependent on the number of feet affected (a maximum of 4 points); visible mouth lesions on the tongue (1 point), gums, lips (1 point), or snout (1 point), with a maximum of 3 points (maximum score = 13). Protection in this study indicated that all regions of pigs, except for the injection site, showed no clinical signs throughout the experimental period (7 days post-challenge). After virus inoculation, levels of virus in nasal discharge and serum samples were monitored for 7 days by collecting the samples at 1-day intervals, and viruses were detected using real-time polymerase chain reaction (RT-PCR) (AccuPower FMDV Real-Time RT-PCR MasterMix Kit; Bioneer, Daejeon, Korea). The clinical scores were based on the sum of each pig’s FMD lesion or signs manifested in each category (maximum score = 13) according to the criteria defined by Alves et al. [14]. The 50% protective dose (PD_50_) values were calculated using the Spearman-Kärber method [11].

### 2.11. Ethical Statement

The animal experiments in this study were approved by the Institutional Animal Care and Use Committee (IACUC) and carried out in accordance with the National Institutes of Health Guide for the care and us of laboratory animals (IACUC approval no. 2020-522).

### 2.12. Statistical Analysis

All data are representative of three independent experiments, and values are represented as the mean  ±  standard deviations (SD). Statistical significance was evaluated with a two-way analysis of variance (ANOVA) followed by Tukey’s or Bonferroni’s post hoc test using GraphPad Prism version 9 (GraphPad Software, La Jolla, CA, USA). Statistical significance was set at *p*  <  0.05, at a 95% confidence interval.

## 3. Results

### 3.1. Determination of Optimal Conditions for Inoculating FMDV onto Suspension Cells

**Figure 1:**
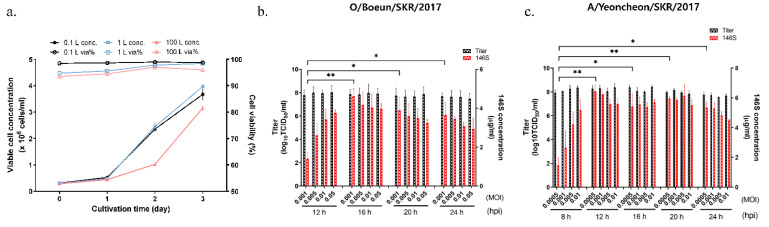
Optimization of the conditions for antigen production in a flask scale. (**a**) Comparison of cell growth curve during cultivation of BHK-21 suspension cell for 3 days in different volumes. Viable cell concentration (filled symbols) and cell viability (empty symbols) were estimated by Vi-cell RX at 0, 1, 2, and 3 days. Determination of optimal virus concentration and harvest time for (**b**) O/Boeun/SKR/2017 and (**c**) A/Yeoncheon/SKR/2017. BHK-21 suspension cells were grown up to 3 × 10^6^ cells/mL for 3 days, inoculated with viruses at different MOIs. The samples were collected at 8 h or 12 h to 24 h post-infection. The data shown are representative of three independent experiments and are presented as the mean ± standard deviations (SD). The asterisks represent the statistical differences: *, *p* < 0.05; **, *p* < 0.005.

As a preliminary test for scale-up of FMD vaccine antigen production, we investigated the optimal conditions for suspension cell culture and virus inoculation. Initially, the rate of cell growth and cell viability were examined in a flask and 100 L bioreactor. The rate of cell growth was lower in the bioreactor (100 L) than in the flasks with no significant difference (Figure 1a). The total cell number in the flask and bioreactor reached 3.5 × 10^6^ cells/mL and 3 × 10^6^ cells/mL, respectively, 3 days after cell seeding at 3 × 10^5^ cells/mL. However, cell viability was maintained at more than 90% throughout the 3 days of cell culture, regardless of the culture volume and equipment.

After various concentrations of O/Boeun/SKR/2017 were inoculated onto BHK-21 suspension cells, the virus supernatant was harvested from 12 to 24 hpi (Figure 1b). While all the titers were above 1.0 × 10^7^ TCID_50_/mL, the 146S antigen content varied depending on the virus concentration and harvest time. At 12 hpi, the amount of 146S antigen increased as the concentration of the virus increased. By contrast, the amount of 146S antigen was inversely proportional to the concentration of the virus from 16 to 24 hpi. Therefore, the optimal conditions for O/Boeun/SKR/2017 were determined to be 0.001 MOI and 16 hpi.

The same experiment was carried out with A/Yeoncheon/SKR/ 2017 (Figure 1c). All virus titers were around 1.0 × 10^8^ TCID_50_/mL. At 8 hpi, the amount of 146S antigen increased as the concentration of the virus increased. From 12 h to 24 h, there was no significant difference depending on the virus concentration. Taken together, the optimal conditions for A/Yeoncheon/SKR/ 2017 were determined to be 0.0005 MOI and 12 hpi.

### 3.2. FMDV Propagation in a 100 L Bioreactor

**Figure 2:**
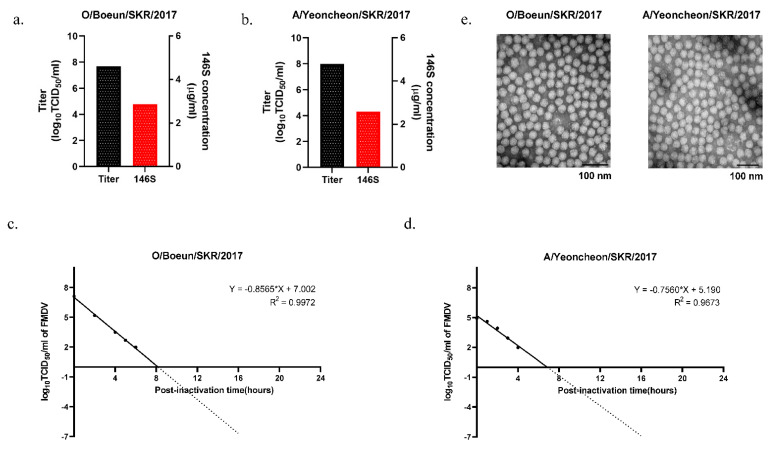
Results of antigen production in a 100 L bioreactor. (**a**,**b**) Contents of 146S antigen and virus titer from virus supernatant produced by a 100 L bioreactor. (**c**,**d**) Virus inactivation kinetics after binary ethyleneimine (BEI) treatment. The virus culture supernatant was treated with 3 mM BEI twice (at 0 h and 16 h) and incubated until 24 h. Samples were taken every hour from the bioreactor for 6 h to measure virus inactivation kinetics for each strain of the virus. The extrapolation of the individual graph is drawn as a linear line for the analysis of FMDV inactivation. (**e**) Inactivated 146S antigen was concentrated with PEG, purified using sucrose gradient ultracentrifugation, and observed by transmission electron microscope (TEM).

O/Boeun/SKR/2017 and A/Yeoncheon/SKR/2017 were propagated in a 100 L bioreactor based on the optimal conditions in a flask. The viruses were harvested at 16 and 12 hpi, respectively, resulting in virus titers being around 1.0 × 10^8^ TCID_50_/mL for both viruses (Figure 2a,b). The amount of 146S antigen was 2.87 µg/mL and 2.59 µg/mL for O/Boeun/SKR/2017 and A/Yeoncheon/SKR/2017, respectively. The inactivation kinetics showed that the slope of the line was linear, and the titers of both O/Boeun/SKR/2017 and A/Yeoncheon/SKR/2017 viruses were less than one infectious particle per 10^4^ L of liquid preparation before the end of the inactivation period (24 h) (Figure 2c,d). After virus inactivation, electron microscopy was performed to determine the physical integrity of the viruses in the process of 100 L bioreactor culture. We could not observe any damage during the scale-up of viruses, with the viruses exhibiting a diameter of 25–30 nm, as expected (Figure 2e).

### 3.3. Immune Responses in Guinea Pigs Vaccinated with the Bivalent Vaccine

**Figure 3:**
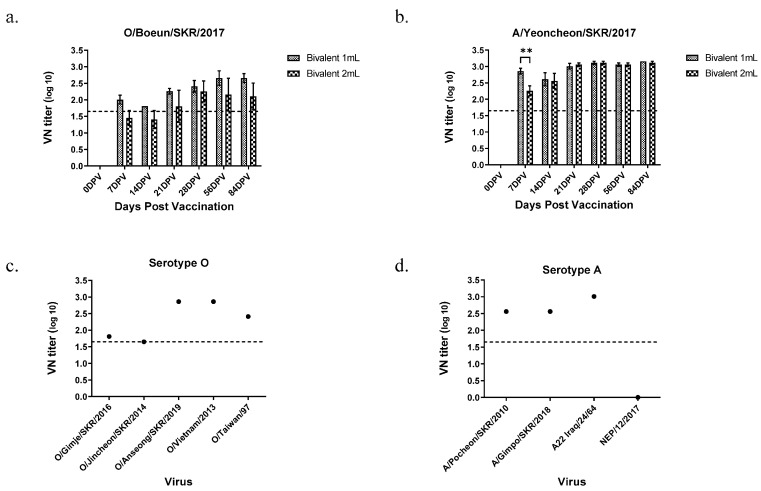
Virus neutralizing antibody titers against homologous and heterologous viruses in guinea pigs after immunization with the bivalent vaccine. Virus neutralization tests for O/Boeun/SKR/2017 (**a**) and A/Yeoncheon/SKR/2017 (**b**) were performed with sera collected from guinea pig vaccinated with two different volumes (1 mL and 2 mL) of the bivalent vaccine until 84 days post-vaccination (dpv). (**c**) Virus neutralization test for the serotype O heterologous viruses was performed with sera collected 28 dpv from guinea pig vaccinated with 1 mL of the bivalent vaccine. (**d**) Virus neutralization test for the serotype A heterologous virus was performed with sera collected 28 dpv from guinea pig vaccinated with 1 mL of the bivalent vaccine. The dotted line represents 1:45 (1.65 log_10_) of virus neutralizing antibody titer that is regarded to be positive according to the OIE terrestrial manual. The data shown are representative of three independent experiments and are presented as the mean ± SD values. The asterisks represent the statistical differences: **, *p* < 0.005.

The final formulated bivalent vaccine contained 15 µg O/Boeun/SKR/2017 and 15 µg A/Yeoncheon/SKR/2017. To minimize granuloma and side effects of the oil adjuvant, we reduced the vaccine volume from 2 mL/dose to 1 mL/dose. The bivalent vaccine was administered to guinea pigs at a volume of 1/10 of 1 mL/dose or 2 mL/dose. The VN titers of sera collected at 7 to 84 dpv for O/Boeun/SKR/2017 were not different between 1 mL/dose and 2 mL/dose (Figure 3a). While the sera at 21 dpv from both 1 mL/dose and 2 mL/dose exhibited VN titers of 1:45 or more for O/Boeun/SKR/2017, the VN titer for the A/Yeoncheon/SKR/2017 was 1:45 or more from 7 dpv and it maintained the same level until 84 dpv regardless of vaccine volume (Figure 3b). Subsequently, VN titers using sera at 28 dpv from 1 mL/dose were measured against type O heterologous viruses (Figure 3c). The VN titers for type O were above 1:45 for all five FMDV strains. The VN titers for type A were also above 1:45 for A/Pocheon/SKR/2010, A22Iraq, and A/Gimpo/SKR/2018, except for A/NEP/12/2017 (Figure 3d).

### 3.4. Protective Dose of the Bivalent Vaccine in Pigs

**Figure 4:**
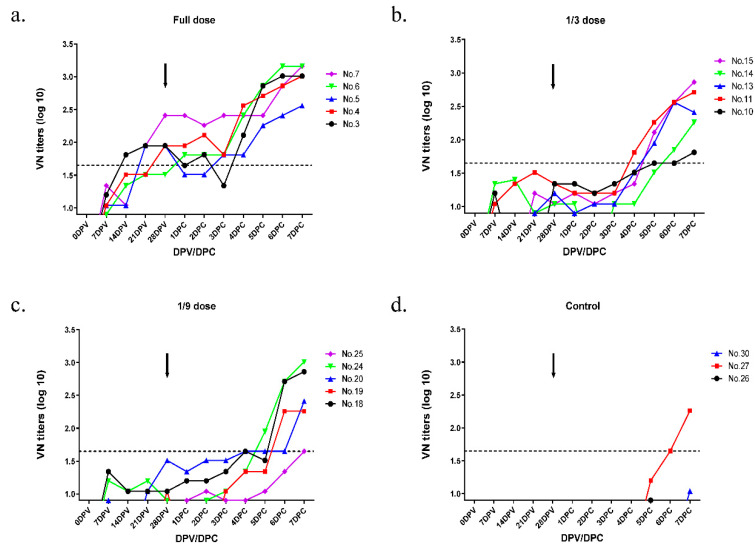
Virus neutralizing antibody titers against serotype O homologous virus in pigs after immunization with the bivalent vaccine. Pigs (*n* = 18) were divided into four groups (full dose, 1/3 dose, 1/9 dose, control) and blood samples were collected at 0, 7, 14, 21, and 28 days post-vaccination. Pigs were challenged with O/Boeun/SKR/2017 at 28 dpv with 1 × 10^5^ TCID_50_/0.1 mL in the bulb of the heel. Blood samples were collected until 7 days post-challenge. Virus neutralization test with O/Boeun/SKR/2017 was performed using the sera collected from vaccinated pigs (five heads per group) with full dose (**a**), 1/3 dose (**b**), and 1/9 dose (**c**). VN titers of the sera were collected from three heads of non-vaccinated pigs as the control (**d**). Black arrows indicate the challenge time at 28 dpv.

**Figure 5:**
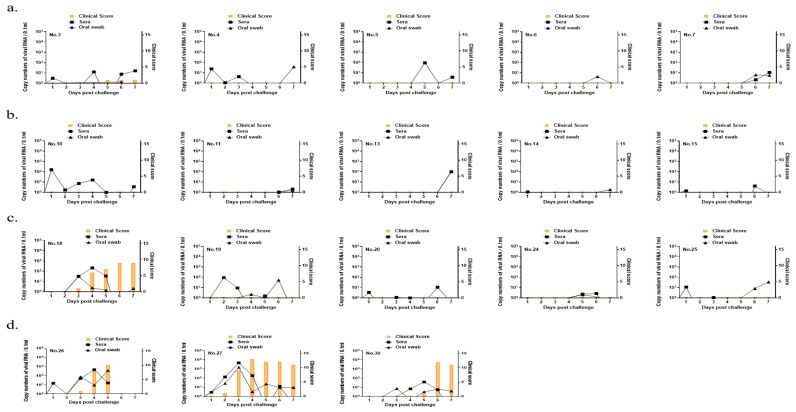
Clinical scores and virus shedding in pigs immunized with a bivalent vaccine after O/Boeun/SKR/2017 challenge. Oral swab and blood samples were collected daily from the pigs (*n* = 18) that were vaccinated with the bivalent vaccine in the full dose (**a**), 1/3 dose (**b**), 1/9 dose (**c**), and control (**d**) groups until 7 days post-challenge. Clinical symptoms were examined daily after the virus challenge. The FMDV RNA was identified by extraction of viral RNA from oral swab samples and quantitative real-time polymerase chain reaction (RT-PCR).

Clinical signs were investigated for 7 days after virus challenge in all immunized groups and the control group. The VN titers for the O/Boeun/SKR/2017 sera were dependent on the antigen concentration in pigs and were highest in the full-dose group (Figure 4). For the O/Boeun/SKR/2017, four pigs in the full-dose group were positive (cutoff of 1.65 log_10_) at 28 dpv (Table 1). We could not observe the clinical score, except for the injection site, in the full-dose and 1/3 dose groups. However, one pig in the 1/9 dose group showed vesicles at several sites (Figure 5). Collectively, the 50% protective dose for the O/Boeun/SKR/2017 bivalent vaccine was 12.5 PD_50_/dose.

**Figure 6:**
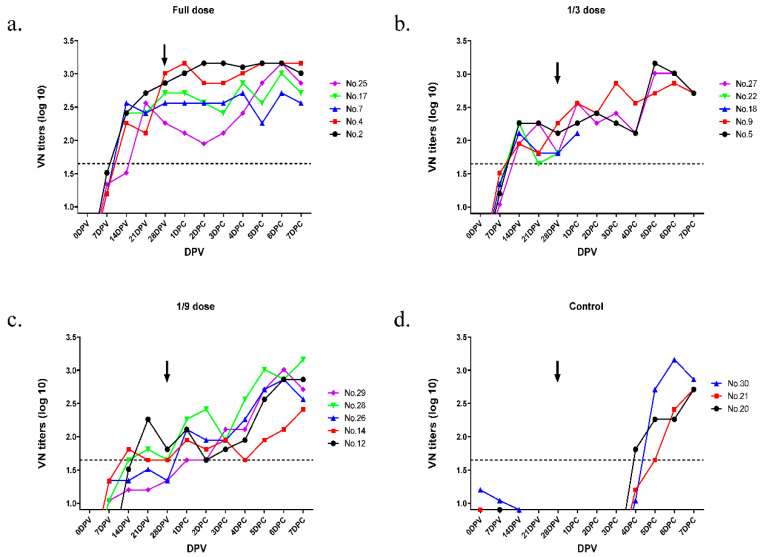
Virus neutralizing antibody titers against serotype A homologous virus in pigs after immunization with the bivalent vaccine. Pigs (*n* = 18) were vaccinated with full dose (**a**), 1/3 dose (**b**), 1/9 dose (**c**), control (**d**) and blood samples were collected and challenged with A/Yeoncheon/SKR/2017 at 28 dpv with 1 × 10^5^ TCID_50_/0.1 mL in the bulb of the heel. Moreover, a virus neutralization test with A/Yeoncheon/SKR/2017 was performed as described in Figure 4. Black arrows indicate the challenge time at 28 dpv.

**Figure 7:**
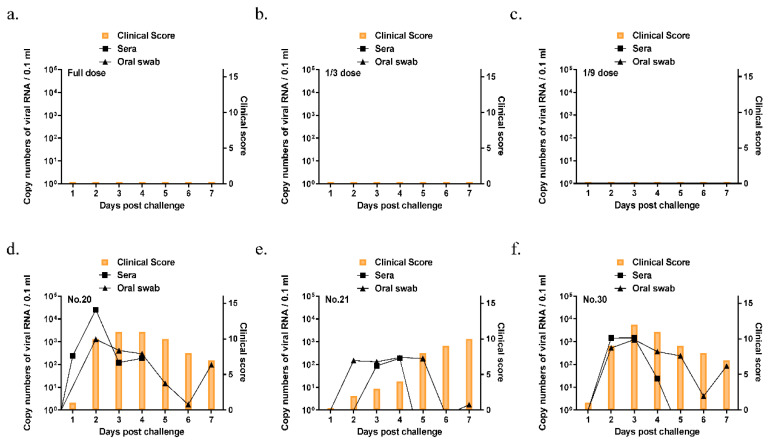
Clinical scores and virus shedding in pigs immunized with a bivalent vaccine after the A/Yeoncheon/SKR/2017 challenge. Oral swab and blood samples were collected daily from the pigs (*n* = 18) that were vaccinated with the bivalent vaccine in the full dose (**a**), 1/3 dose (**b**), 1/9 dose (**c**), and control (**d**–**f**) groups until 7 days post-challenge. Clinical symptoms were examined daily after the virus challenge. The foot-and-mouth disease virus (FMDV) RNA was identified by the extraction of viral RNA from oral swab samples and quantitative real-time polymerase chain reaction (RT-PCR).

For A/Yeoncheon/SKR/2017, all pigs were above 1:45 titer in the full dose and 1/3 dose group at 21 dpv (Figure 6 and Table 2). While the three pigs in the control group showed clinical signs, the clinical score was not observed even at the injection site in all vaccinated groups (Figure 7). Therefore, the 50% protective dose for the A/Yeoncheon/SKR/2017 bivalent vaccine was determined as 15.6 PD_50_/dose.

## 4. Discussion

Although the FMD vaccination policy was implemented across South Korea, FMD outbreaks of serotype O have occurred almost every year since 2014. Additionally, a novel Cathay topotype of serotype O has been reported in neighboring countries [5,15]. The Cathay virus is not known to be matched with the present vaccines [7,16]. FMD outbreaks of serotype A occurred in 2010 and 2017. Therefore, a local FMD vaccine that could cover the type O Cathay strain and is comparable to the A22 IRQ vaccine strain is required in South Korea.

O/Boeun/SKR/2017 and A/Yeoncheon/SKR/2017 were selected as local vaccine strains for industrial production in South Korea. It was also reported that the O/Boeun/SKR/2017 vaccine strain exhibited high immunogenicity and a broad antigenic spectrum for FMDV representative lineages from Asia [8].

The main advantage of suspension culture is its easy expansion by simply increasing the culture volume, enabling the full exploitation of bioreactor capacities up to 20,000 L [17]. The huge experimental cost of FMD vaccine production on an industrial scale makes comprehensive process optimization very challenging. Thus, a scaled-down model representing a large-scale FMD vaccine process is required. In this regard, we attempted to scale up the two vaccine strains up to the pilot scale (100 L) and evaluate the efficacy of the bivalent vaccine before industrial production.

According to a previous report, mammalian cells are prone to damage by turbulent fluid dynamic stress, particularly damage caused by impeller agitation [18]. The cell number in the bioreactor, however, increased 10-fold over 3 days, showing a growth pattern similar to that of the flask scale. After the cells in the bioreactor were cultured for 3 days, they were inoculated with the virus as the viability of cells in the flask dropped gradually after cells reached the maximal number on the 4th day (data not shown). Since the optimal conditions of virus concentration and infection time were different depending on the virus strains, each FMDV strain should be optimized for the virus concentration and infection period for FMD vaccine production.

BHK-21 suspension cells are normally grown in bioreactors for FMD vaccine manufacturing. When the cell density reached approximately 3 million per mL, the proliferated cells were allowed to settle at the bottom of the bioreactor to remove the spent media before virus infection [19]. However, based on a previous report [20], we employed a 30% media addition strategy using Cellvento to make the scale-up process easy and to help save the time and labor involved in spent media removal.

The amount of 146S antigen harvested in a 100 L bioreactor was above 2 µg/mL, the general amount of FMD vaccine antigen in the virus culture supernatant during the FMD vaccine production process [18,21], indicating that the vaccine strains selected in this study were appropriate for FMD vaccine manufacturing.

During the inactivation of the virus, virus samples were collected at hourly intervals to evaluate inactivation kinetics. The inactivation procedure is not considered to be satisfactory unless at least the latter part of the slope of the line is linear and extrapolation indicates that there would be less than one infectious particle per 10^4^ L of liquid preparation at the end of the inactivation period [12]. Both viruses included in the bivalent vaccine met the criteria for virus inactivation kinetics using two inactivation tanks. This was supported by other reports that Indian FMDV strains were also fully inactivated with BEI (1.6 mM) within 8–10 h [22].

The virus cultured in a bioreactor (100 L) exhibited intact morphology without damage and were 25–30 nm in diameter, as observed using electron microscopy, indicating that these two strains were sufficiently stable for commercial mass production. Thus, two viruses were formulated using an adjuvant in a bivalent vaccine. Although the 146S antigens per vaccine dose normally ranged from 1 to 10 µg [23], the 146S antigen content of the experimental vaccine was adjusted to 15 μg/mL, according to a previous study [24].

Currently, commercial FMD vaccines for pigs are formulated using an oil adjuvant. Granulomas have been reported to occur in pigs at injection sites post-vaccination due to the use of oil adjuvants. The issue of injection site granulomas post-vaccination has been particularly highlighted in Korea as the neck region of pigs has significant value in Korean food culture [25]. Therefore, these granulomas cause severe economic losses in pig farms as the lesions in the neck should be discarded during slaughter [26]. In this regard, we tried to minimize the oil adjuvant volume by reducing the vaccine volume from 2 mL/dose to 1 mL/dose [27]. As shown in Figure 3, there was no difference in the immunological response between 1 mL/dose and 2 mL/dose in guinea pigs. However, whether 1 mL/dose can reduce the granuloma compared to 2 mL/dose at 6–7 months of age should be assessed. The homologous VN titers were not different between the two types of volume due to the same amount of 146S. For heterologous viruses, the bivalent vaccine showed a broad spectrum of neutralizing antibody levels of 1:45, which is normally considered to be a cutoff for herd-based serosurveillance [11]. However, the bivalent vaccine did not show sufficient VN titers against the A/Nepal/12/2017 virus. No matching vaccines have been reported for these A/Asia/GVII strains [7].

While vaccines with the potency of 3 PD_50_ are generally suitable for use in routine vaccination campaigns, higher potency vaccines of 6 PD_50_ or more are recommended for their wider spectrum of immunity as well as their rapid onset of protection in the case of an emergency outbreak. In this regard, the local bivalent vaccine developed in this study corresponds to a high-potency vaccine with 10 PD_50_ or more. It is also known that emergency vaccines formulated with high antigen content from vaccine bank antigens often perform better than the results predicted from the in vitro vaccine matching test, and emergency vaccines with vaccine strains that have relatively low r_1_ values against a field strain can provide sufficient heterologous protection [28]. In particular, for A/Yeoncheon/SKR/2017, thirteen pigs in the three groups exhibited positive VN titers (above 1.65 log_10_) at 21 dpv and the clinical score was not observed even at the injection site in all vaccinated groups, indicating that the vaccine could provide sterile protection against the homologous virus.

To the best of our knowledge, this is the first report demonstrating that the vaccine strains could be successfully scaled-up to a 100 L bioreactor, with the determination of its protective efficacy in pigs. This result will shed light on local FMD vaccine production on an industrial scale in the near future.

## Figures and Tables

**Table 1:** Summary of the immune response and protective effect for O/Boeun/SKR/2017 after immunization of pigs with the bivalent vaccine.

Groups	Pig ID	Virus Neutralizing Antibody Titers (log_10_)					Clinical Score	Protection
		0 dpv	7 dpv	14 dpv	21 dpv	28 dpv		
Full dose	#3	<0.9	1.20	1.81	1.95	1.95	1 *	Yes ^a^
	#4	<0.9	1.04	1.51	1.51	1.95	0	Yes
	#5	<0.9	1.04	1.04	1.95	1.95	0	Yes
	#6	<0.9	0.9	1.34	1.51	1.51	0	Yes
	#7	<0.9	1.34	1.04	1.95	2.41	0	Yes
1/3 dose	#10	<0.9	1.20	<0.9	<0.9	1.34	0	Yes
	#11	<0.9	1.04	1.34	1.51	1.34	0	Yes
	#13	<0.9	<0.9	<0.9	0.9	1.2	0	Yes
	#14	<0.9	1.34	1.20	0.9	1.04	0	Yes
	#15	<0.9	<0.9	<0.9	1.2	1.04	0	Yes
1/9 dose	#18	<0.9	1.34	1.04	1.04	1.04	9	No
	#19	<0.9	<0.9	<0.9	<0.9	1.04	0	Yes
	#20	<0.9	0.9	<0.9	1.04	1.51	0	Yes
	#24	<0.9	1.2	1.04	1.20	0.9	0	Yes
	#25	<0.9	<0.9	<0.9	<0.9	<0.9	0	Yes
Control	#26	<0.9	<0.9	<0.9	<0.9	<0.9	10	No
	#27	<0.9	<0.9	<0.9	<0.9	<0.9	13	No
	#30	<0.9	<0.9	<0.9	<0.9	<0.9	12	No

* The lesion at the injection site was counted as the clinical score. ^a^ Vesicle lesion on the site injected with challenge virus.

**Table 2:** Summary of the immune response and protective effect for A/Yeoncheon/SKR/2017 after immunization of pigs with the bivalent vaccine.

Groups	Pig ID	Virus Neutralizing Antibody Titers (log_10_)					Clinical Score	Protection
		0 dpv	7 dpv	14 dpv	21 dpv	28 dpv		
Full dose	#2	<0.9	1.51	2.41	2.71	2.86	0	Yes
	#4	<0.9	1.20	2.26	2.11	3.01	0	Yes
	#7	<0.9	1.20	2.56	2.41	2.56	0	Yes
	#17	<0.9	1.51	2.41	2.41	2.71	0	Yes
	#25	<0.9	1.34	1.51	2.56	2.26	0	Yes
1/3 dose	#5	<0.9	1.20	2.26	2.26	2.11	0	Yes
	#9	<0.9	1.51	1.95	1.81	2.26	0	Yes
	#18	<0.9	1.34	2.11	1.81	1.81	0	Yes
	#22	<0.9	1.34	2.26	1.65	1.81	0	Yes
	#27	<0.9	1.04	1.95	2.26	1.65	0	Yes
1/9 dose	#12	<0.9	<0.9	1.51	2.26	1.81	0	Yes
	#14	<0.9	1.34	1.81	1.65	1.65	0	Yes
	#26	<0.9	1.34	1.34	1.51	1.34	0	Yes
	#28	<0.9	1.04	1.65	1.81	1.65	0	Yes
	#29	<0.9	1.04	1.20	1.20	1.34	0	Yes
Control	#20	<0.9	0.9	<0.9	<0.9	<0.9	11	No
	#21	0.9	<0.9	<0.9	<0.9	<0.9	10	No
	#30	1.2	1.04	0.9	<0.9	<0.9	12	No

## Data Availability

Not applicable.

## Supplementary Materials

The following are available online at https://www.mdpi.com/article/10.3390/vaccines9060586/s1, Figure S1: Phylogenetic analysis of type O (a) and type A (b) FMDV based on the VP1 sequence by MEGA X software with 1000 replicates of bootstrap analysis.

## References

[1] Jamal S.M., Belsham G.J. (2013). Foot-and-mouth disease: Past, present and future. Vet. Res..

[2] Domingo E., Baranowski E., Escarmís C., Sobrino F. (2002). Foot-and-mouth disease virus. Comp. Immunol. Microbiol. Infect. Dis..

[3] Mahapatra M., Upadhyaya S., Aviso S., Babu A., Hutchings G., Parida S. (2017). Selection of vaccine strains for serotype O foot-and-mouth disease viruses (2007-2012) circulating in Southeast Asia, East Asia and Far East. Vaccine.

[4] Blacksell S.D., Siengsanan-Lamont J., Kamolsiripichaiporn S., Gleeson L.J., Windsor P.A. (2019). A history of FMD research and control programmes in Southeast Asia: Lessons from the past informing the future. Epidemiol. Infect..

[5] Vierra D., Bertram M.R., Palinski R.M., Pauszek S.J., Hartwig E.J., Smoliga G.R., Vu L.T., Hoang B.H., Phuong N.T., Hung V.V. (2020). Foot-and-mouth disease virus serotype O/CATHAY genome sequences from five outbreaks in Vietnam, 2017 to 2019. Microbiol. Res. Announc..

[6] Singanallur N.B., Dekker A., Eble P.L., van Hemert-Kluitenberg F., Weerdmeester K., Horsington J., Vosloo W.W. (2020). Emergency foot-and-mouth disease vaccines A Malaysia 97 and A22 Iraq 64 offer good protection against heterologous challenge with A variant serotype A ASIA/G-IX/SEA-97 lineage virus. Vaccines.

[7] Mahapatra M., Parida S. (2018). Foot and mouth disease vaccine strain selection: Current approaches and future perspectives. Expert Rev. Vaccines.

[8] Lee G., Hwang J.H., Park J.H., Lee M.J., Kim B., Kim S.M. (2020). Vaccine strain of O/ME-SA/Ind-2001e of foot-and-mouth disease virus provides high immunogenicity and broad antigenic coverage. Antiviral Res..

[9] Lee S.-Y., Lee Y.-J., Kim R.-H., Park J.-N., Park M.-E., Ko M.-K., Choi J.-H., Chu J.-Q., Lee K.-N., Kim S.-M. (2017). Rapid engineering of foot-and-mouth disease vaccine and challenge viruses. J. Virol..

[10] Spearman C. (1908). The method of ‘right and wrong cases’ (‘constant stimuli’) without Gauss’s formulae. Br. J. Psychol..

[11] Kärber G. (1931). Beitrag zur kollektiven Behandlung pharmakologischer Reihenversuche. Naunyn-Schmiedebergs Archiv für experimentelle Pathologie und Pharmakologie.

[12] International Committee, Biological Standards Commission, International Office of Epizootics (2018). Manual of Diagnostic Tests and Vaccines for Terrestrial Animals: Mammals, Birds, and Bees.

[13] Doel T.R., Baccarini P.J. (1981). Thermal stability of foot-and-mouth disease virus. Arch. Virol..

[14] Alves M.P., Guzylack-Piriou L., Juillard V., Audonnet J.C., Doel T., Dawson H., Golde W.T., Gerber H., Peduto N., McCullough K.C. (2009). Innate immune defenses induced by CpG do not promote vaccine-induced protection against foot-and-mouth disease virus in pigs. Clin. Vaccine Immunol..

[15] Brito B., Pauszek S.J., Hartwig E.J., Smoliga G.R., Vu L.T., Dong P.V., Stenfeldt C., Rodriguez L.L., King D.P., Knowles N.J. (2018). A traditional evolutionary history of foot-and-mouth disease viruses in Southeast Asia challenged by analyses of non-structural protein coding sequences. Sci. Rep..

[16] Horsington J., Zhang Z., Bittner H., Hole K., Singanallur N.B., Alexandersen S., Vosloo W. (2015). Early protection in sheep against intratypic heterologous challenge with serotype O foot-and-mouth disease virus using high-potency, emergency vaccine. Vaccine.

[17] Gallo-Ramirez L.E., Nikolay A., Genzel Y., Reichl U. (2015). Bioreactor concepts for cell culture-based viral vaccine production. Expert Rev. Vaccines.

[18] Li X.R., Yang Y.K., Wang R.B., An F.L., Zhang Y.D., Nie J.Q., Ahamada H., Liu X.X., Liu C.L., Deng Y. (2019). A scale-down model of 4000-L cell culture process for inactivated foot-and-mouth disease vaccine production. Vaccine.

[19] Genzel Y., Vogel T., Buck J., Behrendt I., Ramirez D.V., Schiedner G., Jordan I., Reichl U. (2014). High cell density cultivations by alternating tangential flow (ATF) perfusion for influenza A virus production using suspension cells. Vaccine.

[20] Kim A.Y., Kim H., Park S.Y., Park S.H., Lee J.M., Kim J.S., Park J.W., Park C.K., Park J.H., Ko Y.J. (2021). Investigation of the optimal medium and application strategy for foot-and-mouth disease vaccine antigen production. J. Appl. Microbiol..

[21] Barteling S.J., Meloen R.H. (1974). A simple method for the quantification of 140 s particles of foot-and-mouth disease virus (FMDV). Archiv für die gesamte Virusforschung.

[22] Aarthi D., Ananda Rao K., Robinson R., Srinivasan V.A. (2004). Validation of binary ethyleneimine (BEI) used as an inactivant for foot and mouth disease tissue culture vaccine. Biologicals.

[23] Doel T.R. (2003). FMD vaccines. Virus Res..

[24] Park J.-N., Lee S.-Y., Chu J.-Q., Lee Y.-J., Kim R.-H., Lee K.-N., Kim S.-M., Tark D.-S., Kim B., Park J.-H.J.V. (2014). Protection to homologous and heterologous challenge in pigs immunized with vaccine against foot-and-mouth disease type O caused an epidemic in East Asia during 2010/2011. Vaccine.

[25] Lyons N.A., Lyoo Y.S., King D.P., Paton D.J. (2016). Challenges of generating and maintaining protective vaccine-induced immune responses for foot-and-mouth disease virus in pigs. Front. Vet. Sci..

[26] Ko E.Y., Jung S., Jeong H.K., Han J.H., Son J.H. (2018). Effects of foot-and-mouth disease vaccination location and injection device on the incidence of site lesions in pork. Korean J. Food Sci. Anim. Resour..

[27] Park M.-E., You S.-H., Lee S.-Y., Lee K.-N., Ko M.-K., Choi J.-H., Kim B., Lee J.-S., Park J.-H. (2017). Immune responses in pigs and cattle vaccinated with half-volume foot-and-mouth disease vaccine. J. Vet. Sci..

[28] Dekker A., Sanz-Bernardo B., Singanallur N.B., Ludi A.B., Horsington J., Eble P.L., King D.P., Vosloo W. (2020). Cross-protection induced by a A/MAY/97 emergency vaccine against intra-serotype heterologous challenge with a foot-and-mouth disease virus from the A/ASIA/G-VII lineage. Vaccines.

