# Supplementary material for: Scale-Up Production of Type O and A Foot-and-Mouth Disease Bivalent Vaccine and Its Protective Efficacy in Pigs
Source: Vaccines (Basel). 2021 Jun 2;9(6):586. doi: 10.3390/vaccines9060586 (PMC8227705; doi:10.3390/vaccines9060586)
Supplement: Supplementary file 1 [file vaccines-09-00586-s001.zip › vaccines-1192548-supplementary.pdf]

a.

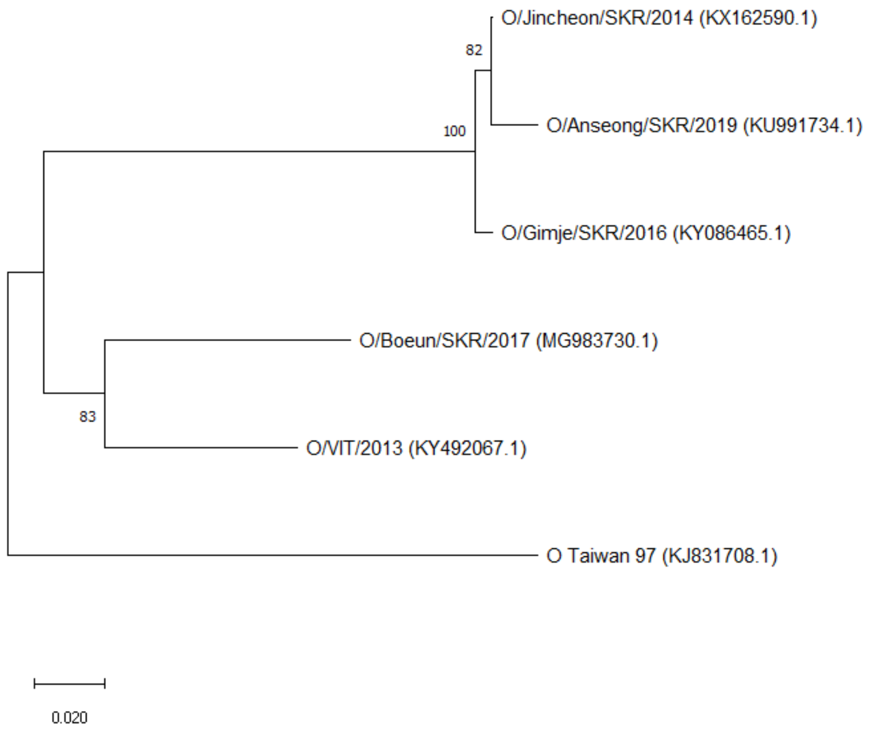

b.

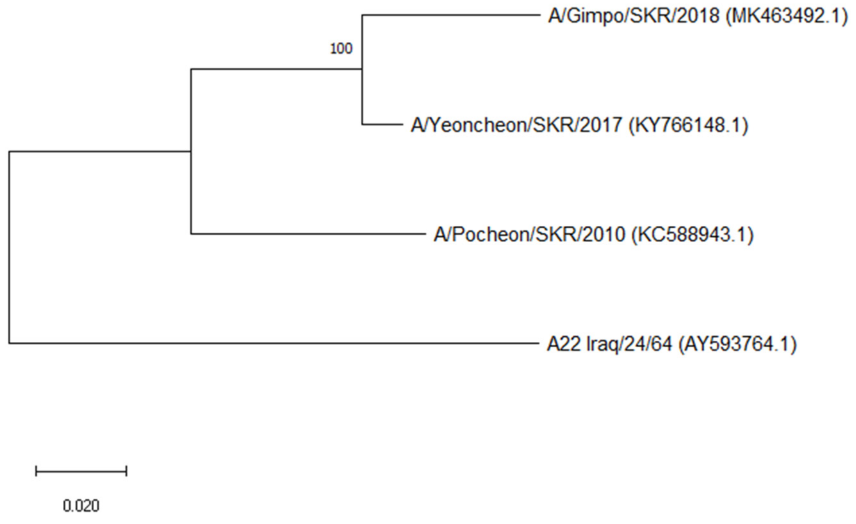

Figure S1. Phylogenetic analysis of type O (a) and type A (b) FMDV based on the VP1 sequence by MEGA X software with 1,000 replicates of bootstrap analysis.
